# Supplementary material for: Advanced Optical Microscopy: Unveiling Functional Insights Regarding a Novel PPP2R1A Variant and Its Unreported Phenotype
Source: Int J Mol Sci. 2023 Sep 5;24(18):13699. doi: 10.3390/ijms241813699 (PMC10530954; doi:10.3390/ijms241813699)
Supplement: Supplementary file 1 [file ijms-24-13699-s001.zip › ijms-2543317-supplementary.pdf]

Advanced optical microscopy: unveiling the functional insights regarding a novel *PPP2R1A* variant and its unreported phenotype

Supplementary Material.

Suppl. Table S1: List of pathogenic mutations and their related phenotype.

cDNA	protein	Reported phenotype*	HEAT	Reference
c.61C>T	p.R21C	PCH, mild ID	1	This work
c.96C>G	p.I32M	NDD	1	Lenaerts (2021) [7]
c.421T>A	p.F141I	NDD	4	Lenaerts (2021) [7]
c.455C>T	p.S152F	NDD	4	Lenaerts (2021) [7]
c.533C>A	p.T178N	NDD	5	Lenaerts (2021) [7]
c.532A>T	p.T178S	NDD	5	Lenaerts (2021) [7]
c.536C>T	p.P179H	NDD and CHD	5	Baker (2022) [8]
c.536C>T	p.P179L	NDD	5	Houge (2015) [1]; Lenaerts (2021) [7]
c.539T>G	p.M180R	NDD	5	Lenaerts (2021) [7]
c.539T>A	p.M180K	NDD	5	Lenaerts (2021) [7]
c.539T>C	p.M180T	NDD	5	Lenaerts (2021) [7]
c.538A>G	p.M180V	NDD	5	Lenaerts (2021) [7]
c.544C>T	p.R182W	NDD ± CHD	5	Houge (2015) [1]; Lenaerts (2021) [7]; Baker (2022) [8]; Lei (2023) [9]
c.548G>A	p.R183Q	Ventriculomegaly, severe NDD ± CHD	*5*	Wallace (2019) [5]; Baker (2022) [8]
c.547C>T	p.R183W	NDD	5	Lenaerts (2021) [7]
c.656C>T	p.S219L	Developmental & epileptic encephalopathy	6	Zhang (2020) [6]; Lenaerts (2021) [7]
c.658G>A	p.V220M	NDD	6	Lenaerts (2021) [7]
c.773G>A	p.R258H	NDD/ID	7	Houge (2015) [1]; Lenaerts (2021) [7]
c.772C>A	p.R258S	NDD	7	Lenaerts (2021) [7]

* According to HGMD Pro. Accessed June 2023. CHD: Congenital heart disease; ID: Intellectual disability; NDD Neurodevelopmental disorder; PCH Pontocerebellar Hypoplasia

```
                                                                                       Arg21
PPP2R1A_Human                               --------------------------MAAADGDDSLYPIAVLIDELRNEDVQLRLNSIKK    34
Macaca_mulatta_Mammal                       --------------------------MAAADGDDSLYPIAVLIDELRNEDVQLRLNSIKK    34
Mus_musculus_Mammal                         --------------------------MAAADGDDSLYPIAVLIDELRNEDVQLRLNSIKK    34
Camelus_ferus_Mammal                        --------------------------MAAADGDDSLYPIAVLIDELRNEDVQLRLNSIKK    34
Aotus_nancymaae_Mammal                      --------------------------MAAADGDDSLYPIAVLIDELRNEDVQLRLNSIKK    34
Loxodonta_africana_Mammal                   --------------------------MAAADGDDSLYPIAVLIDELRNEDVQLRLNSIKK    34
Bos_taurus_Mammal                           --------------------------MAAADGDDSLYPIAVLIDELRNEDVQLRLNSIKK    34
Xenopus_tropicali_Amphibian                 --------------------------MAAADGDDSLYPIAVLIDELRNEDVQLRLNSIKK    34
Anolis_carolinensis_Reptilia                --------------------------MAAADGDDSLYPIAVLIDELRNEDVQLRLNSIKK    34
Gekko_japonicus_Reptilia                    --------------------------MAAADGDDSLYPIAVLIDELRNEDVQLRLNSIKK    34
Python_bivittatus_Reptilia                  --------------------------MAAADGDDSLYPIAVLIDELRNEDVQLRLNSIKK    34
Pipra_filicauda_Birds                       --------------------------MAAADGDDSLYPIAVLIDELRNEDVQLRLNSIKK    34
Taeniopygia_guttata_Birds                   --------------------------MAAADGDDSLYPIAVLIDELRNEDVQLRLNSIKK    34
Catharus_ustulatus_Birds                    --------------------------MAAADGDDSLYPIAVLIDELRNEDVQLRLNSIKK    34
Takifugu_rubripes_Fish                      --------------------------MAGADGDDSLYPIAVLIDELRNEDVQLRLNSIKK    34
Rhincodon_typus_Fish                        -----------------------MAAAEVADGDDSLYPIAVLIDELRNEDVQLRLNSIKK    37
Amblyraja_radiata_Fish                      -----------------------MAAAEVADGDDSLYPIAVLIDELRNEDVQLRLNSIKK    37
Orchesella_cincta_Insect                    ------------------------MATTETCGDESLYPIAVLIDELRNEDVQLRLNSIKK    36
Zootermopsis_nevadensis_Insect              ------------------------MAASDSSGDDSLYPIAVLIDELKNEDVQLRLNSIKK    36
Tpd3p_Saccharomyces_cerevisiae_Yeast        MSGARSTTAGAVPSAATTSTTSTTSNSKDSDSNESLYPLALLMDELKHDDIANRVEAMKK    60
Violaceomyces_palustris_Mushroom            -----------------------------MDSDEQLYPIAILIDELKSDDVTLRLNAIHR    31
Melanopsichium_pennsylvanicum_Mushroom      -----------------------------MEADEQLFPIAILIDELKSDDVTLRLNAIHR    31
Pseudomicrostroma_glucosiphilum_Mushroom    -----------------------------MDQDDQLYPIAILIDELKSDDVTLRLNAIHR    31
                                                                            ::.*:*:*:*:***: :*:  *::::::
```

Suppl. Figure S1. PPP2R1A protein multiple alignment. N-terminal fragment of the human PPP2R1A and orthologues showing high conservation of the residues, including Arg21, present in all vertebrates and the presence of Lys, with similar properties, in other organisms.

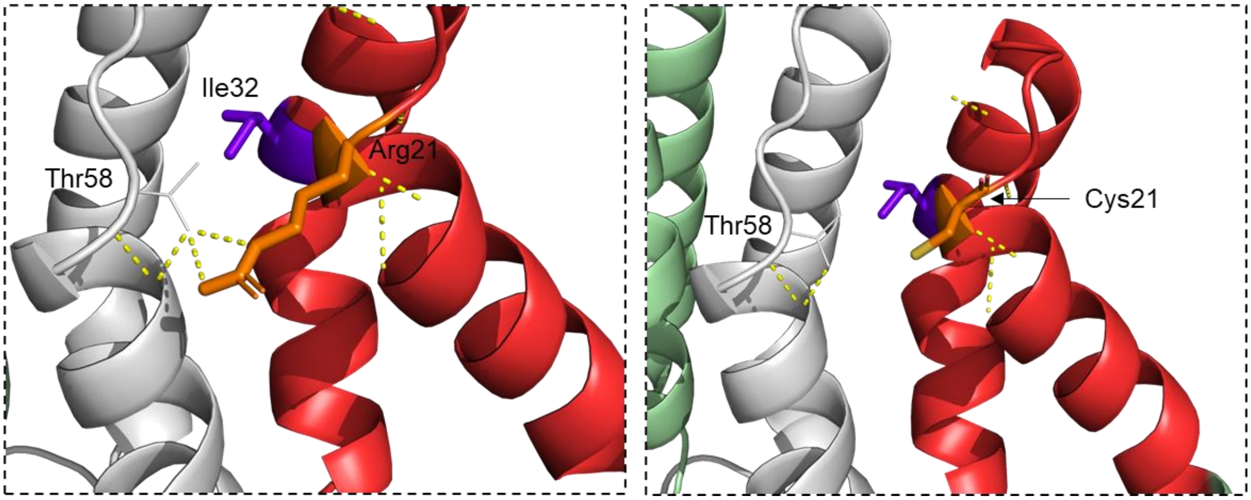

Suppl. Figure S2. *In silico* study of HEAT1 tertiary structure. Closeup of the mutated HEAT1 (red) region showing Arg21 (left) hydrogen bonds with the lateral chain of Thr58 at HEAT2 (white). These interactions are not properly formed in the presence of cysteine (right).
